# The impacts of knowledge, risk perception, emotion and information on citizens’ protective behaviors during the outbreak of COVID-19: a cross-sectional study in China

**DOI:** 10.1186/s12889-020-09892-y

**Published:** 2020-11-23

**Authors:** Liangwen Ning, Jinyu Niu, Xuejing Bi, Chao Yang, Ze Liu, Qunhong Wu, Ning Ning, Libo Liang, Aishu Liu, Yanhua Hao, Lijun Gao, Chaojie Liu

**Affiliations:** 1 School of Health Management, Harbin Medical University, Harbin, China; 2 Harbin Center for Disease Control and Prevention, Harbin, China; 3 School of Education Science, Harbin Normal University, Harbin, China; 4 School of Psychology and Public Health, La Trobe University, Melbourne, VIC 3086 Australia

**Keywords:** COVID-19, China, Behavior, Public health, Perception

## Abstract

**Background:**

Individual protective behaviors play an important role in the control of the spread of infectious diseases. This study aimed to investigate the adoption of protective behaviors by Chinese citizens amid the COVID-19 outbreak and its associated factors.

**Methods:**

An online cross-sectional survey was conducted from 22 January to 14 February 2020 through Wenjuanxing platform, measuring their knowledge, risk perception, negative emotion, response to official communication, and protective behaviors in relation to COVID-19. A total of 3008 people completed the questionnaire, of which 2845 were valid questionnaires.

**Results:**

On average, 71% of respondents embraced protective behaviors. Those who made no error in the knowledge test (AOR = 1.77, *p* < 0.001) perceived the high severity of the epidemic (AOR = 1.90, *p* < 0.001), had high negative emotion (AOR = 1.36, *p* = 0.005), reported good health (AOR = 1.94, *p* < 0.001), paid high attention to the governmental media (AOR = 4.16, *p* < 0.001) and trusted the governmental media (AOR = 1.97, *p* < 0.001) were more likely to embrace protective behaviors after adjustments for variations in potential confounding factors. Women and older people were also more likely to embrace protective behaviors. No regional or educational differences were found in the adoption of protective behaviors.

**Conclusion:**

The majority of Chinese citizens embraced protective behaviors. Higher levels of protective behaviors are associated with higher knowledge, perceived severity, negative emotion, and attention to and trust in the official governmental media. Official governmental communication is the largest single predictor of protective behaviors.

**Supplementary Information:**

The online version contains supplementary material available at 10.1186/s12889-020-09892-y.

## Background

Coronavirus Disease 2019 (COVID-19) is one of the most serious respiratory illnesses caused by the severe acute respiratory syndrome coronavirus 2 (SARS-CoV-2) since the 1918 global influenza pandemic. It has had a profound health and economic impact on the world and shows little sign of containment yet [1]. On 30 January 2020, the World Health Organization (WHO) declared the COVID-19 outbreak a public health emergency of international concern [2]. The outbreak has not only resulted in a serious death toll, but also triggered a range of social and psychological reactions, imposing immeasurable consequences on society.

Non-pharmaceutical interventions have been regarded as the most effective measures to end the pandemic in the absence of an effective vaccine and medical treatment at the moment, which require the public to adopt protective behaviors [3, 4]. These include preventive measures (such as social distancing, hand hygiene, and wearing face masks), avoidance measures (such as home isolation and quarantine and travel restriction), and illness management measures (such as medical consultation, testing, and infection control) [5]. Studies have also shown that these measures can have the positive psychological effect and protect mental health [6–8]. These measures are particularly important and challenging in densely populated communities [9]. The WHO encourages everybody to take responsibility and adopt protective behaviors [10]. But each country may weigh these measures differently taking into consideration the social and cultural context of the country. While social distancing and hand hygiene have been widely accepted as the most important strategies to control the outbreak of COVID-19 [1], the effectiveness of wearing masks by the public has attracted some debate [11]. Empirical evidence shows that restricting crowd gathering in public places can reduce influenza infection by 23% [12]. Hand hygiene practices were also proved to be equally effective in reducing the transmission of SARS-CoV, another virus from the coronavirus family [13]. Most Asian countries, including China, have encouraged the public to wear face masks in public places from the very beginning of the battle against COVID-19. But it has been endorsed only recently by the US and some European countries.

Success or otherwise of the non-pharmaceutical interventions depends largely on the acceptance of the public, although some measures can be made compulsory by law [5]. The unprecedented lockdown measures taken by the Chinese government stunned the world. Researchers tend to attribute China’s success in the control over the outbreak of COVID-19 to such strong governmental actions [14]. But limited attention has been paid to public responses to the government’s actions. Understanding how the public behaves is critical for the government to develop effective communication strategies and ensure high compliance to protective behaviors. However, there is a dearth of literature documenting the protective behaviors of Chinese citizens amid the outbreak of COVID-19. In this study, we selected the items of protective behaviors based on the Guidelines for the Prevention and Control of COVID-19 issued by the Chinese Centers for Disease Control and Prevention. The guidelines clearly recommend that the public should gather less, wear masks when going out and so on. Previous studies on public knowledge and attitudes toward the outbreak of infectious diseases were usually conducted over the recovery stage [15]. It is important to note that both governmental policies and behaviors of the public are likely to vary and change over time depending on the situation of the epidemic. This study aimed to fill the gap in the literature by assessing the protective behaviors adopted by citizens in China during the middle of the outbreak of COVID-19. The study also explored the determinants of the protective behaviors based on the knowledge-attitudes-practices (KAP) model.

## Methods

### Study setting

A cross-sectional questionnaire survey of citizens in China over the period from 22 January to 14 February 2020 was conducted when the epicenter Wuhan was placed in complete lockdown. During this period, all provinces launched a category one emergency response, the harshest response designed to control the most serious public health events such as plague and anthrax. In China, public health emergency responses are classified into four levels ranging from the most restrictive to the least restrictive. A category one response involves the strictest measures, including travel restrictions, bans on public events and gatherings, the closure of schools/universities and non-essential services, and isolation and quarantine requirements. The provinces in which a category one response was declared have to follow commands and control from the central government. The four-level system also envisages a gradual staggering of relaxed measures over the recovery period. Local governments regain higher levels of autonomy when emergency responses are downgraded.

### Study participants

Adults over the age of 18 years in China were eligible to participate in this study. The survey was voluntary and anonymous. The return of completed questionnaire was deemed as informed consent.

### Data collection

The questionnaire survey was conducted online, the only available avenue for data collection at the time. An online survey has the advantage of reaching a large audience rapidly at the cost of bias toward those who have access to the online platform [16]. The study used Wenjuanxing, a widely accepted online questionnaire survey platform in China for data collection.

This research uses the sample service function of the Wenjuanxing platform. The platform has a large number of potential sample population to ensure the randomness of sampling and the reliability of inferences. We require the platform to invite respondents of different age groups and places of citizens to participate the survey to ensure the sample better representative.

The survey started with an explanation of the purpose and protocol of the study. Participants had to read and confirm their agreement with the statement before proceeding to the questionnaire. They could withdraw at any time whilst completing the questionnaire. Only those surveys without any missing answers could be submitted. Each respondent was allowed to submit one questionnaire only, according to the IP address recorded by Wenjuanxing.

A total of 5579 people accessed the online survey and 3008 (53.9%) completed the questionnaire. The research team performed a logic check on the returned questionnaires and excluded 163 containing logic errors, which resulted in a final sample size of 2845 (94.5% of the returned questionnaires) for data analyses.

### Measurements

The survey was guided by the theories of the *Social Amplification of Risk Framework (SARF)* [17] *and* the *Knowledge, Attitude and Practices (KAP)* behavioral model [18], measuring five constructs: knowledge, risk perception, negative emotion, official communication, and protective behaviors in relation to COVID-19. (Additional file 1). The questionnaire items were either derived from the WHO guidelines [19] or adapted from the existing measurement scales [20]. The questionnaire also captured the sociodemographic characteristics of the respondents. The language of the survey is Chinese.

#### Knowledge

Appropriate knowledge is a prerequisite condition for the public to take action [21]. A lack of understanding is a major obstacle to public compliance with emergency response measures [9]. According to the *KAP* model, changes in human behavior occur in three stages: knowledge acquisition, belief generation, and behavior formation [18]. Appropriate knowledge not only encourages the public to take action, but also facilitates the right action. Empirical evidence on the importance of knowledge in the prevention and control of infectious diseases has been found in many countries [22] across a range of diseases including COVID-19. This survey contained 11 knowledge questions, asking what COVID-19 is (e.g. is it a kind of influenza), what symptoms are associated with it (e.g. fatigue, dry cough, fever, difficulty in breathing), how it is transmitted (e.g. through droplets), and how it can be prevented (e.g. hand hygiene, face masks). We did not ask what to do if infected because at the time, it was governmental policy that all infected cases were isolated and treated in hospitals (including makeshift hospitals) in China. The knowledge test questions were aligned with the WHO guidelines. Answers to these questions can be found on the website of the Chinese Centers for Disease Control and Prevention (http://www.chinacdc.cn/jkzt/crb/zl/szkb_11803/) and the National Health Commission of People’s Republic of China (http://www.nhc.gov.cn/). During the epidemic, the Chinese government carried out knowledge popularization through television, websites, social media, community advocacy and so on. These intensive publicity has made the Chinese public generally have a high level of knowledge on COVID-19. In order to better distinguish the level of the public’s knowledge on COVID-19, respondents were categorized into two groups: those who gave correct answers to all of the questions were classified as high level group and the others as relative lower level group.

#### Risk perception

Risk perception refers to individual’s intuitive risk assessment, reflecting public attitudes or beliefs about potential harm [23]. It is widely accepted that perceived risk is fundamental for triggering behavioral changes [16]. Those who downplay the potential harm of a risk event are less likely to take targeted actions to prevent the event [23]. Risk perception was found to be a predictor of behavioral changes amid the outbreak of H1N1 influenza and SARS [15, 24]. Risk perception is usually shaped by one’s knowledge. Knowledge enables the proper self-assessment of the risk of an event and its associated consequences [25]. Several studies have proved that individuals who perceive a high risk [26] and high severity [27] of the COVID-19 outbreak are most likely to take precautionary action. However, researchers are increasingly concerned about the potential bias of risk perception in the public when high levels of uncertainty remain for COVID-19, a new disease for which our understanding is limited [26]. Biased risk perception can make behavioral decisions deviate from rationality, resulting in serious social and economic consequences [28]. Several broadly applicable scales exist which measure risk perception based on a consensus that risk perception is a multidimensional concept [20]. Most scales consider it to be a function of individual affective reaction to the possibility and consequentiality of risks. The *SARF* theory extends the dimension to include interactions between individuals and social contexts, incorporating psychological, social and cultural perspectives into the measurement of risk perception [17]. This is because a particular social environment may amplify or attenuate individual responses to the risk or risk event. The risk perception scale we used in this study is introduced from “Public Risk Perception Scale for Public Health Emergencies” we developed in 2018. The scale has gone through a rigorous development process and has been published in the Chinese Journal of Public Health [29]. Reliability and validity analysis results show that the scale has good reliability (Cronbach’s α = 0.75) and validity (GFI = 0.982, AGFI = 0.963, IFI = 0.947, TLI = 0.914, RMSEA = 0.059). Expert consultations and pilot survey were carried out to ensure the quality of the questionnaire. In this study, three components of risk perception were measured, namely susceptibility (3 items), severity (4 items), and controllability (2 items). Example questions include “I am very likely to be infected” (susceptibility); “The spread of COVID-19 is very wide” (severity); “It is difficult to treat” (controllability). Study participants were asked to rate each question on a five-point Likert scale ranging from 1 “strongly disagree” to 5 “strongly agree”. A summed average score for each component was calculated. A score above 3 was deemed as high in risk perception.

#### Emotional response

High levels of mental health problems have been observed in China during the COVID-19 outbreak accompanied by prolonged social isolation [7]. Fear of COVID-19 is believed to be one of the central factors elevating the level of stress and anxiety [30]. Although fear is a negative emotional response, it may exacerbate perceived risks [31], stimulating protective behaviors [32]. Fear has long been recognized to have an “*inverted U-shaped drive function*” [33]. It can prompt individuals to solve the problem or avoid the problem [34]. Fear may increase people’s alertness to potential risks, triggering a stronger willingness to avoid the risk. But if a negative emotional response lasts too strong, it can also prevent people from engaging in certain protective behaviors [35]. Measuring the emotional responses of people in social isolation during the COVID-19 outbreak has started to attract attention from the research community [7]. But unfortunately, no valid scale was available when we collected the data. In this study, we designed three items measuring fear (I am afraid that I or my family will be infected with COVID-19), worry (I am very worried when I know someone who is coming back from or going to Wuhan), and psychological tension (I am very nervous about the epidemic). Study participants were asked to rate their emotional response on a five-point Likert scale ranging from 1 “strongly disagree” to 5 “strongly agree”. A summed average score above 3 was deemed as high in negative emotional responses.

#### Official communication

Information communication is critical for the public to obtain knowledge and comply with or defy governmental advice. Balkhy et al. [36] found that public attention to the media played an essential role in the control of swine flu in Saudi Arabia through behavioral changes. Public media provides a platform for individuals to interact with the broader society*,* which can shape their attitudes toward governmental policies. Media use has a profound impact on knowledge acquisition at a time of crisis [37], especially when the entire society is virtually locked down. Meanwhile, media reports can also fuel fear [38]. Modern information technology has broadened the source of information dramatically. While it enables easy and rapid access to information, the increased diversity of information also brings great challenges to government communications with the public. In a public health crisis like COVID-19, consistency in information communications is critical. In some countries, governments have been frustrated with the media [39]. Indeed, trust plays a critical role in the public acceptance of government messages. Under a high level of uncertainty due to limited knowledge, the public are likely to simply follow the agencies they trust [40]. In this study, we measured public attention to and trust in the official governmental media in relation to COVID-19. Study participants were asked to rate their experience on a five-point Likert scale ranging from 1 “never” to 5 “always”. A score above 3 was deemed as high in attention or trust.

#### Protective behaviors

The protective behaviors assessed in this study were drawn from the research of Bish [5] and the guidelines issued by the Chinese government. These included avoiding crowds, wearing face masks, keeping good indoor airflow, maintaining overall health, following official guidelines, encouraging behavioral compliance of other people, preventing spread of the virus to others, practicing good hand hygiene, and avoiding contact with wild animals. Study participants were asked to rate their behaviors on a five-point Likert scale ranging from 1 “never” to 5 “always”. A summed average score was calculated, with a score above 3 indicating a high level of embracement of protective behaviors.

The sociodemographic data captured in this study included location, residency (urban vs rural), gender, age, marital status, educational attainments, perceived health, and vulnerability of family members to COVID-19. Previous studies showed that these sociodemographic characteristics are associated with public knowledge and protective behaviors in relation to various infectious diseases [5]. According to the official reports, Hubei (with Wuhan as capital city), Guangdong, Zhejiang, Henan, Hunan, Anhui, Jiangxi and Chongqing were considered high-risk areas in China. By 14 February, 61,475 cases and 1486 deaths were reported in the high-risk areas, compared with a total of 5101 cases and 38 deaths in other regions in China. Pregnant women, the elderly (≥65 years) and children (≤5 years) were commonly considered as the vulnerable populations, deserving extra protection from society [41]. In this study, self-reported health was rated on a five-point Likert scale ranging from 1 (very poor) to 5 (very good).

### Statistical analysis

The characteristics of the study participants were described through frequency analyses and the respondents from high-risk areas were compared with those from other regions using Chi-square tests.

The percentage of correct answers given to the knowledge questions was calculated for each item and all items combined, respectively. Knowledge differences between the respondents from high-risk areas and other regions were compared using Chi-square tests.

The mean value and standard deviation (SD) of the average summed score for each risk perception component and the protective behaviors were calculated and compared between the respondents from high-risk areas and other regions using student *t* tests.

The participants were categorized into two groups: those who had a summed average score above 3 in protective behaviors (highly protective) and those who did not. Logistic regression models were established to determine the predictors of the protective behaviors. Variables in relation to the sociodemographic characteristics of the participants and their self-ratings on knowledge, risk perception, negative emotion, and official communication were entered into the regression models using an enter approach.

To test the robustness of the findings of the logistic regression model, a linear regression model was established with the protective behavior scores being treated as a continuous variable. Partial least squares structural equation modeling (SEM) analyses were performed to verify the theoretical hypotheses that guided the selection of the measurements in this study. In the SEM, knowledge, risk perception and official communication were treated as formative measurements while emotional response and protective behaviors were treated as reflective measurement [42]. The SEM results supported all of the theoretical hypotheses (Additional File 2).

All analyses in this study were performed using SPSS/Win and SmartPLS. A *p* value of less than 0.05 was considered statistically significant.

## Results

### Demographic characteristics of respondents

Urban citizens accounted for 48.6% of the study sample. About 32.2% lived in the high-risk areas. The majority of respondents were female (58.2%) and single (54.0%). Most were younger than 40 years (85.6%), had a university degree (69.1%), lived with a vulnerable family member (71.4%), and reported good health (73%). The respondents from the high-risk areas were younger and more likely to be male and resided in rural areas compared with those from other regions (Table 1).

**Table 1:** Sociodemographic Characteristics of Respondents

Characteristics	Total		High-Risk Areas		Other Regions		***p***
	***n*** = 2845	%	***n*** = 915	%	***n*** = 1930	%	
**Residency**							< 0.001
Rural	1462	51.4	579	63.3	883	45.8	
Urban	1383	48.6	336	39.6	1047	54.2	
**Gender**							0.028
Male	1188	41.8	409	44.7	779	40.4	
Female	1657	58.2	506	55.3	1151	59.6	
**Age (years)**							< 0.001
< 25	1204	42.3	399	43.6	805	41.7	
25–40	1233	43.3	419	45.8	814	42.2	
> 40	408	14.3	97	10.6	311	16.1	
**Education**							< 0.001
No university degree	879	30.9	337	36.8	542	28.1	
University degree	1966	69.1	578	63.2	1388	71.9	
**Marital status**							0.189
Not married	1535	54.0	510	55.7	1025	53.1	
Married	1310	46.0	405	44.3	905	46.9	
**Living with vulnerable people**							0.758
No	813	28.6	258	28.2	555	28.8	
Yes	2032	71.4	657	71.8	1375	71.2	
**Self-reported health**							0.351
Poor	769	27.0	237	25.9	532	27.6	
Good	1073	73.0	678	74.1	1398	72.4	

### COVID-19 knowledge of respondents

The percentage of correct answers given to the 11 questions ranged from 54.1 to 97.6% (Table 2). On average, the respondents gave a correction answer to 9.7 (SD = 1.46) questions. About 34.5% answered all of the questions correctly. More than 90% of respondents understood the symptoms of COVID-19 and its transmission routes and preventive measures. No differences in knowledge between those from the high-risk areas and other regions were found (Table 2).

**Table 2:** COVID-19 knowledge of respondents

Question	Respondents answering questions correctly						***p***
	Total		High-risk Area		Other Regions		
	n	%	n	%	n	%	
**What is COVID-19?**							
COVID-19 is different from influenza	1540	54.1	474	51.8	1066	55.2	0.086
There is no special treatment, but many symptoms can be managed	2152	75.6	699	76.4	1453	75.3	0.520
**Symptoms of COVID-19?**							
Generally include fever (some not), fatigue, dry cough, and dyspnea	2757	96.9	892	97.5	1865	96.6	0.219
Most have mild to moderate symptoms, but a few serious even die	2185	76.8	694	75.8	1491	77.3	0.406
**Transmission route?**							
COVID-19 can be transmitted from person to person	2734	96.1	882	96.4	1848	95.8	0.417
COVID-19 virus can spread through breathing and droplets	2730	96.0	880	96.2	1854	96.1	0.885
**Preventive measures?**							
Avoid crowded places	2721	95.6	878	96.0	1843	95.5	0.571
Wear a face mask in public places	2728	95.9	886	96.8	1842	95.4	0.081
Wash hands frequently	2777	97.6	892	97.5	1885	97.7	0.767
Maintain regular ventilation at home	2638	92.7	850	92.9	1788	92.6	0.808
Early detection, diagnosis, treatment and isolation	2658	93.4	856	93.6	1802	93.4	0.853
**All questions**	982	34.5	314	34.3	668	34.6	0.877

### Risk perception of respondents

A high level of the perceived severity of the COVID-19 outbreak was found, with 94.4% respondents giving a rating higher than 3 out of a possible score of 5. Slightly more than half (58.2%) of the respondents weren’t optimistic (with a score > 3) about the controllability of the outbreak. There was a relatively low perception of susceptibility, with only 29.2% respondents giving a rating higher than 3 out of a possible score of 5. No differences in perceived controllability were found between those from the high-risk areas and other regions. But the respondents from the high-risk areas had higher scores in perceived susceptibility and severity compared with their counterparts from other regions (Table 3).

**Table 3:** Risk Perception of Respondents

Dimension	Risk perception scores (Mean ± SD)				Risk perception score > 3 [N (%)]			
	Total	High-risk Area	Other Region	***p***	Total	High-risk Area	Other Region	***p***
**Susceptibility**	**2.76 ± 0.76**	**2.88 ± 0.75**	**2.71 ± 0.76**	**< 0.001**	**830(29.2)**	**323 (35.3)**	**507 (26.3)**	**< 0.001**
I am very likely to be infected	2.35 ± 0.98	2.36 ± 0.98	2.35 ± 0.98	0.660	345 (12.1)	113 (12.3)	232 (12.0)	0.802
I will be infected if in the same room with a patient	3.43 ± 1.11	3.44 ± 1.11	3.43 ± 1.11	0.698	1543 (54.3)	500 (54.6)	1043 (54.0)	0.763
The epidemic is serious in my community	2.50 ± 1.11	2.83 ± 1.18	2.35 ± 1.05	0.000	527 (18.5)	257 (28.1)	270 (14.0)	< 0.001
**Severity**	**4.25 ± 0.62**	**4.30 ± 0.59**	**4.23 ± 0.63**	**0.002**	**2685 (94.4)**	**870 (95.1)**	**1815 (94.0)**	**0.260**
The spread of COVID-19 is very wide	4.43 ± 0.82	4.50 ± 0.77	4.39 ± 0.83	0.000	2527 (88.8)	834 (91.1)	1693 (87.7)	0.007
The outbreak is very serious	4.30 ± 0.82	4.38 ± 0.78	4.56 ± 0.69	0.000	2456 (86.4)	821 (89.7)	1635 (84.7)	< 0.001
It has high mortality	4.13 ± 0.94	4.18 ± 0.90	4.11 ± 0.95	0.076	2253 (79.2)	745 (81.4)	1508 (78.1)	0.044
Health impact is very serious if infected	4.15 ± 0.85	4.16 ± 0.86	4.15 ± 0.84	0.789	2363 (83.1)	767 (83.8)	1596 (82.7)	0.453
**Controllability**	**3.37 ± 0.87**	**3.38 ± 0.84**	**3.37 ± 0.89**	**0.717**	**1655 (58.2)**	**551 (60.2)**	**1104 (57.2)**	**0.128**
It is difficult to treat	3.53 ± 0.96	3.54 ± 0.96	3.53 ± 0.97	0.908	1642 (57.7)	526 (57.5)	1116 (57.8)	0.865
The spread of COVID-19 is difficult to control	3.21 ± 1.09	3.22 ± 1.06	3.20 ± 1.10	0.641	1209 (42.5)	390 (42.6)	819 (42.4)	0.925

### Protective behaviors

Overall, 71.0% of respondents obtained a summed average behavior score above 3, indicating a high level of embracement of protective behaviors. Relatively higher levels of actions for protecting others (e.g. stay away from others if ill) and compliance with official advice were found, compared with the measures of protecting oneself such as hand hygiene and health maintenance. The respondents from high-risk areas were more likely to avoid crowds than those from other regions. But the respondents outside the high-risk areas appeared to be keener to maintain physical health and avoid contact with wild animals (Table 4).

**Table 4:** Protective Behaviors of Respondents

Protective Behaviors	Mean ± SD of behavior scores				N (%) with a behavior score > 3			
	Total	High-risk areas	Other Regions	***p***	Total	High-risk Areas	Other Regions	***p***
Avoid crowds	3.97 ± 1.12	4.05 ± 1.15	3.93 ± 1.11	0.005	2074 (72.9)	703 (76.8)	1371 (71.0)	0.001
Wear face mask	3.97 ± 1.23	4.01 ± 1.23	3.95 ± 1.24	0.269	2042 (71.8)	668 (73.0)	1374 (71.2)	0.315
Open house windows	4.01 ± 1.02	4.03 ± 1.04	4.01 ± 1.02	0.644	2158 (75.9)	699 (76.4)	1459 (75.6)	0.642
Maintain physical health	3.77 ± 1.03	3.71 ± 1.04	3.79 ± 1.03	0.037	1791 (63.0)	556 (60.8)	1235 (64.0)	0.096
Follow official advices	4.13 ± 0.98	4.14 ± 0.97	4.13 ± 0.98	0.865	2303 (80.9)	748 (81.7)	1555 (80.6)	0.455
Remind family members and friends to take precautions	4.24 ± 0.96	4.24 ± 0.97	4.24 ± 0.97	0.973	2388 (83.9)	768 (83.9)	1620 (83.9)	0.998
Stay away from others if infected	4.31 ± 1.02	4.31 ± 1.02	4.32 ± 1.01	0.805	2389 (84.0)	762 (83.3)	1627 (84.3)	0.488
Wash hands frequently and stop touching face	4.07 ± 1.02	4.00 ± 1.03	4.11 ± 1.01	0.009	2169 (76.2)	685 (74.9)	1484 (76.9)	0.235
Avoid contact with wild animals	4.47 ± 1.04	4.44 ± 1.08	4.50 ± 1.02	0.143	2477 (87.1)	778 (85.0)	1699 (88.0)	0.026
Total	4.11 ± 0.77	4.10 ± 0.76	4.11 ± 0.77	0.833	2021 (71.0)	653 (71.4)	1368 (70.9)	0.790

### Factors associated with preventative behaviors

Female and older respondents were more likely to adopt protective behaviors. Those who were married and had a university degree appeared to be more likely to adopt protective behaviors. But the differences became statistically insignificant after adjustments for variations in other variables. Higher levels of knowledge (AOR = 1.77, *p* < 0.001) and perceived severity (AOR = 1.90, *p* < 0.001) were associated with higher levels of protective behaviors. Perceived susceptibility and controllability did not show significant associations with protective behaviors. Those who reported good health (AOR = 1.94, *p* < 0.001) and high negative emotion (AOR = 1.36, *p* = 0.005) were more likely to adopt protective behaviors. Attention (AOR = 4.16, *p* < 0.001) to and trust (AOR = 1.97, *p* < 0.001) in official governmental media were proved to be an independent predictor of protective behaviors (Table 5). Similar results were found in the linear regression analyses (Additional File 3).

**Table 5:** Factors associated with protective behaviors

Predictor	Number (%) of respondents with a summed average behavior score > 3	Unadjusted		Adjusted			
		OR	***p***	OR	95% Confidence Interval		***p***
**Gender**							
Male (Reference)	802 (67.5)						
Female	1219 (73.6)	1.34	< 0.001	1.21	1.00	1.44	0.040
**Age** (years)			0.001				0.009
< 25 (Reference)	811 (67.4)						
25–40	901 (73.1)	1.32	0.002	1.31	1.01	1.69	0.040
> 40	30 (75.7)	1.51	0.002	1.79	1.25	2.58	0.002
**Education**							
< University (Reference)	1424 (72.4)						
University	597 (67.9)	1.24	0.014	1.11	0.91	1.35	0.298
**Marital status**							
Not married (Reference)	1043 (67.9)						
Married	978 (74.7)	1.39	< 0.001	1.03	0.79	1.34	0.832
**Area**							
Other region (Reference)	1368 (70.9)						
High-risk area	653 (71.4)	1.02	0.790	1.03	0.85	1.25	0.756
**Knowledge**							
Lower level (Reference)	1232 (66.1)						
High level	789 (80.3)	2.09	< 0.001	1.77	1.46	2.15	< 0.001
**Perceived susceptibility**							
≤ 3 (Reference)	1417 (70.3)						
> 3 (High)	604 (72.8)	1.13	0.191	1.01	0.73	1.38	0.966
**Perceived severity**							
≤ 3 (Reference)	78 (48.8)						
> 3 (High)	1943 (72.4)	2.75	< 0.001	1.90	1.54	2.34	< 0.001
**Perceived controllability**							
≤ 3 (Reference)	851 (71.5)						
> 3 (High)	1170 (70.7)	0.96	0.635	1.03	0.85	1.24	0.769
**Negative emotion**							
≤ 3 (Reference)	379 (62.2)						
> 3 (High)	1642 (73.4)	1.68	< 0.001	1.36	1.10	1.68	0.005
**Self-reported health**							
≤ 3 (Reference)	451 (58.6)						
> 3 (Good)	1570 (75.6)	2.19	< 0.001	1.94	1.61	2.34	< 0.001
**Information attention**							
≤ 3 (Reference)	32 (20.5)						
> 3 (High)	1989 (74.0)	3.69	< 0.001	4.16	2.74	6.32	< 0.001
**Trust in official media**							
≤ 3 (Reference)	162 (49.7)						
> 3 (High)	1859 (73.8)	2.85	< 0.001	1.97	1.52	2.56	< 0.001

**Note:**Unadjusted OR was based on unary logistic regression, then incorporate all variables into the model to produce adjusted results

## Discussion

Overall, the adoption level of protective behaviors in this study population is high and similar to the public responses to previous outbreaks of other infectious diseases. On average, 71% of respondents obtained a summed average score higher than 3 indicating a high level of embracement of the protective behaviors, with each item ranging from 63.0% (maintain physical health) to 87.1% (avoid contact with wild animals). Understandably, those behaviors that have a limited disruption on daily routines are more likely to be adopted than the others. One of the interesting findings of this study is that the respondents appeared to be quite mindful of the possibility of infecting others. A higher percentage of respondents embraced altruistic behaviors, such as self-isolation when falling ill (84.0%), following official advice (80.9%) and reminding others (83.9%), compared with the behaviors of protecting oneself from infection, such as hand hygiene (76.2%) and wearing a face mask for self-protection (71.8%). But we cannot rule out the possibility of the potential response bias of the study participants. People tend to deny behaviors that can harm others. Despite difficulties in comparing behaviors across populations and studies due to variations in disease contexts and measurement tools, it appears that the adoption level of the protective behaviors in this study population is similar to that of the Hong Kong population during the 2003 SARS epidemic. Lau et al. [43] found that 65–87% of Hong Kong citizens endorsed hand hygiene, wearing a face mask, and household disinfection guidelines to prevent SARS. But only 24–75% of Hong Kong citizens tried to avoid crowds, far below the level (73%) revealed in this study. This is likely to be a result of lower levels of concern about the potential SARS infection transmitted by asymptomatic carriers. In addition, keeping physical distance in densely-populated cities is always a great challenge. Setbon and colleagues [44] also found that during the 2009 influenza outbreak in France, people were more likely to maintain hand hygiene and avoid contact with patients who had cold symptoms (55–67%) than the blanket avoidance of crowded places (13–27%). In comparison with these findings, the level of reported behaviors of crowd avoidance in this study population is deemed relatively high. It was more so in the high-risk areas, perhaps due to the unprecedented harsh lockdown measures taken by the government.

Although knowledge was found to be an independent predictor of protective behaviors, it offers a limited explanation of the level of protective behaviors in the respondents. On average, the study participants gave a correct answer to 9.7 (88%) questions out of a total of 11. Such a level of knowledge is high, which is consistent with the findings of another study conducted in China [22]. However, the 34.5% of respondents who answered all of the questions correctly have only slightly higher odds (AOR = 1.77, *p* < 0.001) than the others of adopting the protective behaviors.

Of the three components of risk perception, only perceived severity was found to have a significant association, albeit at a low level (AOR = 1.90, *p* < 0.001), with the adoption of protective behaviours. Most respondents considered the outbreak severe (94.4%) but close to half of the respondents considered it controllable (41.8%). But only 29.2% had a perception of high susceptibility. Kwok [45] found that 89% citizens in Hong Kong felt susceptible to COVID-19 and 97% considered the outbreak serious. The low level of perceived susceptibility in this study population may be associated with their young age. This may also explain why the altruistic behaviors were highly appreciated by the study participants. Previous studies showed that a strong perception of the seriousness of a pandemic and its health consequences is an independent predictor of protective behaviors [46]. This was proved to be true during the outbreak of the Middle East Respiratory Syndrome (MERS) and H1N1 flu [46, 47]. Researchers believe that a higher level of risk perception can stimulate anxiety and stress, which encourages the adoption of coping strategies [48]. Those who are more aware of the serious health consequences are more willing to maintain vigilance and take active actions. However, Wachinger et al. [49] noticed that high risk perception is not always associated with high preparedness for natural disasters.

Negative emotion is also an independent predictor of protective behaviors (AOR = 1.36, *p* = 0.005). Researchers found that respondents with a moderate level of anxiety were more likely to adopt precautionary measures during the 2003 SARS-CoV epidemic [50]. Wang and Tee found that specific precautionary measures were associated with lower levels of psychological responses during the COVID-19 outbreak [6–8]. According to Harper’s study [32], fear had a functional role and was a predictor of positive behavior change. Consistently, our finding suggests that negative emotion was associated with protective behaviors and may help to keep general public safe during COVID-19 pandemic.

Attention to the official governmental media was found to be the largest independent predictor of protective behaviors. The odds of the respondents adopting protective behaviors is 4.16 times greater for those who paid a high-level of attention to the official governmental media than those who paid low attention. This may be a result of the highly “command and control” approach to the control of the COVID-19 outbreak. The Chinese culture is usually characterized by collectivism. Compliance with societal orders is high [51]. Previous studies have also demonstrated the importance of information communication. The impact of information on the cognition and emotion of the public is called by some scholars the *exposure effect* [52]. It can shape the view of the public, creating a mood of engagement in crisis management.

One of the prerequisite conditions of the *exposure effect* is the trust of the public in the source of information [53]. Indeed, trust was found to be an independent predictor of protective behaviors. The odds of embracing protective behaviors by those who trusted in the official governmental media is almost double those who had low trust in the official governmental media. This result is consistent with the findings of previous research [5]. Menon et al. [54] concluded that transparency, trustfulness, and communication strategies were important measures for the Singaporean government to employ to guide the public to effectively respond to the SARS crisis. In a pandemic such as COVID-19, governments should maintain authenticity in information disclosure, preferably as the sole source of truth. This is particularly important when the public has access to such a wide range of sources of information through social media. Researchers have found that detailed and true health information can effectively curb info-epidemics and gain public trust while outdated health information, wrong information may increase the risk of serious consequences [55, 56]. Once the public has lost trust in the official governmental media, they are more likely to be misled by false information and produce erroneous risk assessment, resulting in inappropriate behaviors. Slovic [53] points out that trust is fragile and difficult to maintain. Negative events can easily undermine trust. Therefore, the official governmental information has to be open, timely and honest [57].

Consistent with other studies [58], we found that women and older people are more likely to embrace protective behaviors than their male and younger counterparts. Previous studies found that women and older people felt more vulnerable and had a stronger sense of responsibility and willingness to protect society [59]. This study also found that good self-reported health is associated with protective behaviors. But such a link cannot be assumed as causal. The direction of the link may go either way.

### Limitations

This study has some limitations. Although we have used the sample service function to expand the sample ratio of the elderly and rural population, the online survey sample is not representative for the entire population in China. The elderly and those with low levels of education are less likely to have access to the online survey platform than others. Although the study established associations between protective behaviors and knowledge, risk perception and official communication, no casual relationships should be assumed due to the nature of the cross-sectional design. Future research may need to consider the problem of data correction of online survey samples to make it more representative and longitudinal research to explore the relationship between variables.

## Conclusion

The behavioral responses of the public to COVID-19 in China are similar to those of the previous outbreaks of infectious diseases, with the majority embracing protective measures. Higher levels of protective behaviors are associated with high knowledge, perceived severity, negative emotion, and attention to and trust in the official governmental media. Official governmental communication is the largest single predictor of protective behaviors.

## Supplementary Information

**Additional file 1:** Questionnaire. Questionnaire developed for this research

**Additional file 2: Table S1**. Constructs and items measured in the PLS SEM. **Table S2**. Psychometric Table of Measurements. **Fig. S1**. Results of SEM. **Table S3**. Path coefficients tested in the SEM

**Additional file 3: Table S4**. Results of linear regression models

## Data Availability

The datasets generated and analyzed during the current study are not publicly available because the datasets are currently used for another project, but are available from the corresponding author on reasonable request.

## Abbreviations

COVID-19: Coronavirus disease 2019
WHO: World health Organization
SARS-CoV-2: Severe acute respiratory syndrome coronavirus 2
KAP: Knowledge, attitude and practices
SARF: Social amplification of risk framework
SEM: Structural equation model
