# Supplementary material for: The impacts of knowledge, risk perception, emotion and information on citizens’ protective behaviors during the outbreak of COVID-19: a cross-sectional study in China
Source: BMC Public Health. 2020 Nov 23;20:1751. doi: 10.1186/s12889-020-09892-y (PMC7681179; doi:10.1186/s12889-020-09892-y)
Supplement: Supplementary file 1 — Additional file 1: Questionnaire. Questionnaire developed for this research [file 12889_2020_9892_MOESM1_ESM.docx]

**Additional file 1:**

**Questionnaire. Questionnaire developed for this research**

**Questionnaire on Risk Perception and Behavior of Public Health Emergencies**

***We really appreciated for your participating in this survey. To understand the public perception, emotion, knowledge, official communication and behaviors related to the COVID-19 pandemic and to analyze the relevant influencing factors to improve the active response to public health crisis. We sincerely hope to know your opinions and thoughts to the pandemic. All information that you provided will be kept strictly confidential and only used for academic research with your consent. Your support and cooperation will be highly appreciated.***

1. **Socio-demographic information**

A1. Place of abode: ________________

A2. Type of place of residence: 🞏Urban 🞏Rural

A3. Gender: 🞏Male 🞏Female

A4. Age ____

A5. Marital status: 🞏Unmarried 🞏Married 🞏Married 🞏Divorce

🞏Death of a spouse 🞏Others

A6. Education level: 🞏Illiteracy 🞏Elementary 🞏Junior 🞏Senior/technical school

🞏Technical secondary school 🞏College. 🞏Undergraduate course

🞏Master or above

A7. Do you have a child under 5 years old in your family? 🞏Yes 🞏No

A8. Do you have an adult over 65 years older in your family? 🞏Yes 🞏No

A9. Is there a pregnant woman in your family? 🞏Yes 🞏No

A10. Generally speaking, how is your health? 🞏Very poor 🞏Poor 🞏Moderate

🞏Good 🞏Very good

1. **Protective behaviors**

The following questions are about protective behaviors during this pandemic. Choose that best one which fits your personal status.

|  | never | rarely | sometimes | often | always |
| --- | --- | --- | --- | --- | --- |
| Avoid crowds | 🞏 | 🞏 | 🞏 | 🞏 | 🞏 |
| Wear face mask | 🞏 | 🞏 | 🞏 | 🞏 | 🞏 |
| Open house windows | 🞏 | 🞏 | 🞏 | 🞏 | 🞏 |
| Maintain physical health | 🞏 | 🞏 | 🞏 | 🞏 | 🞏 |
| Follow official advices | 🞏 | 🞏 | 🞏 | 🞏 | 🞏 |
| Remind family members and friends to take precautions | 🞏 | 🞏 | 🞏 | 🞏 | 🞏 |
| Stay away from others if infected | 🞏 | 🞏 | 🞏 | 🞏 | 🞏 |
| Wash hands frequently and stop touching face | 🞏 | 🞏 | 🞏 | 🞏 | 🞏 |
| Avoid contact with wild animals | 🞏 | 🞏 | 🞏 | 🞏 | 🞏 |

1. **Knowledge**

| C1. Which of the following statement about COVID-19 is correct? [Select ones if they are applied] |
| --- |
| 🞏 COVID-19 is different from influenza |
| 🞏 There is no special treatment, but many symptoms can be managed |
| C2. Which of the following statement about symptoms of COVID-19 is correct? [Select ones if they are applied] |
| 🞏 Generally include fever (some not), fatigue, dry cough, and dyspnea |
| 🞏 Most have mild to moderate symptoms, but a few serious even die |
| C3. Which of the following statement about transmission route of COVID-19 is correct? [Select ones if they are applied] |
| 🞏 COVID-19 can be transmitted from person to person |
| 🞏 COVID-19 virus can spread through breathing and droplets |
| C4. Which of the following statement about preventive measures of COVID-19 is correct? [Select ones if they are applied] |
| 🞏 Avoid crowded places |
| 🞏 Wear a face mask in public places |
| 🞏 Wash hands frequently |
| 🞏 Maintain regular ventilation at home |
| 🞏 Early detection, diagnosis, treatment and isolation |

1. **Risk perception**

The following questions are about risk perception of the COVID-19 pandemic. Please choose an option that best fits your true feelings about the pandemic.

|  | strongly disagree | disagree | unsure | agree | strongly agree |
| --- | --- | --- | --- | --- | --- |
| I am very likely to be infected | 🞏 | 🞏 | 🞏 | 🞏 | 🞏 |
| I will be infected if in the same room with a patient | 🞏 | 🞏 | 🞏 | 🞏 | 🞏 |
| The epidemic is serious in my community | 🞏 | 🞏 | 🞏 | 🞏 | 🞏 |
| The spread of COVID-19 is very wide | 🞏 | 🞏 | 🞏 | 🞏 | 🞏 |
| The outbreak is very serious | 🞏 | 🞏 | 🞏 | 🞏 | 🞏 |
| It has high mortality | 🞏 | 🞏 | 🞏 | 🞏 | 🞏 |
| Health impact is very serious if infected | 🞏 | 🞏 | 🞏 | 🞏 | 🞏 |
| It is difficult to treat | 🞏 | 🞏 | 🞏 | 🞏 | 🞏 |
| The spread of COVID-19 is difficult to control | 🞏 | 🞏 | 🞏 | 🞏 | 🞏 |

1. **Emotional Response**

The following questions are about Emotional Response of the COVID-19 pandemic. Please choose an option that best fits your true feelings about the pandemic

|  | strongly disagree | disagree | unsure | agree | strongly agree |
| --- | --- | --- | --- | --- | --- |
| I am afraid that I or my family will be infected with COVID-19 | 🞏 | 🞏 | 🞏 | 🞏 | 🞏 |
| I am very worried when I know someone who is coming back from or going to Wuhan | 🞏 | 🞏 | 🞏 | 🞏 | 🞏 |
| I am very nervous about the epidemic | 🞏 | 🞏 | 🞏 | 🞏 | 🞏 |

1. **Official communication**

F1. Have you been following the COVID-19 outbreak from the official governmental media?

🞏 Never 🞏 Rarely 🞏 Sometimes 🞏 Often 🞏 Always

F2. Do you trust in the official governmental media in relation to COVID-19?

🞏 Never 🞏 Rarely 🞏 Sometimes 🞏 Often 🞏 Always
