# Supplementary material for: The impacts of knowledge, risk perception, emotion and information on citizens’ protective behaviors during the outbreak of COVID-19: a cross-sectional study in China
Source: BMC Public Health. 2020 Nov 23;20:1751. doi: 10.1186/s12889-020-09892-y (PMC7681179; doi:10.1186/s12889-020-09892-y)
Supplement: Supplementary file 2 — Additional file 2: Table S1. Constructs and items measured in the PLS SEM. Table S2. Psychometric Table of Measurements. Fig. S1. Results of SEM. Table S3. Path coefficients tested in the SEM [file 12889_2020_9892_MOESM2_ESM.docx]

**Additional file 2:**

**Table S1.** Constructs and items measured in the PLS SEM.

**Table S2.** Psychometric Table of Measurements.

**Figure S1.** Results of SEM.

**Table S3.** Path coefficients tested in the SEM

**Table S1.** Constructs and items measured in the PLS SEM

| **Construct** | **Items** | **Description** |
| --- | --- | --- |
| **Protective Behavior** | B1 | I try not to go to crowded public places, such as restaurants, department stores and public transportation to reduce the chance of infection |
|  | B2 | I wear a mask to reduce the risk of getting infected |
|  | B3 | I open windows in my house and office |
|  | B4 | I maintain physical health consciously through various means |
|  | B5 | I follow the official behavioral guidelines |
|  | B6 | I remind my family members and friends to take precaution measures |
|  | B7 | I try my best to stay away from relatives, friends, and colleagues if infected |
|  | B8 | I try to wash my hands frequently and stop touching my eyes, mouth and nose |
|  | B9 | I try my best to avoid any contacts with wild animals |
| **Risk perception** | R1 | I am very likely to be infected |
|  | R2 | I will be infected if a patient is in the same room as me |
|  | R3 | I think the epidemic is serious in my community |
|  | R4 | I think the spread of this disease is very wide |
|  | R5 | I think this outbreak is very serious |
|  | R6 | COVID-19 has a high mortality rate |
|  | R7 | The infection has very serious impacts on health |
|  | R8 | I think COVID-19 is difficult to treat |
|  | R9 | I think the outbreak is difficult to control |
| **Negative Emotion** | E1 | I worry that I or my family members will be infected |
|  | E2 | I am very nervous about the outbreak |
|  | E3 | I am very concerned when I know someone coming back from or going to Wuhan |
| **Knowledge** | K1 | What is COVID-19? |
|  | K2 | What the symptoms of COVID-19? |
|  | K3 | What are the transmission routes? |
|  | K4 | What preventive measures work? |
| **Official Communication** | O1 | Attention to official governmental media |
|  | O2 | Trust in official governmental media |

Knowledge, risk perception and official communication were treated as a formative measurement, while negative emotion and protective behavior were treated as a reflective measurement. The Cronbach's Alpha (CA), Composite Reliability (CR) and Average Variance Extracted (AVE) coefficients indicate that the reflective measurements are appropriate for the modelling (Table S2).

**Table S2.** Psychometric Table of Measurements

| **Reflective Construct** | **Item** | **Loading** | **Standard Error** | **t** | **p** |
| --- | --- | --- | --- | --- | --- |
| **Protective Behavior**  CA=0.896  CR=0.914  AVE=0.543 | B1 | 0.704 | 0.016 | 44.218 | 0.000 |
|  | B2 | 0.722 | 0.014 | 51.508 | 0.000 |
|  | B3 | 0.740 | 0.013 | 57.223 | 0.000 |
|  | B4 | 0.599 | 0.020 | 30.111 | 0.000 |
|  | B5 | 0.783 | 0.014 | 56.551 | 0.000 |
|  | B6 | 0.818 | 0.009 | 88.687 | 0.000 |
|  | B7 | 0.762 | 0.013 | 58.648 | 0.000 |
|  | B8 | 0.725 | 0.015 | 47.669 | 0.000 |
|  | B9 | 0.758 | 0.015 | 51.601 | 0.000 |
| **Negative Emotion** |  |  |  |  |  |
| CA=0.618  CR=0.794  AVE=0.563 | E1 | 0.784 | 0.013 | 60.048 | 0.000 |
|  | E2 | 0.716 | 0.020 | 36.605 | 0.000 |
|  | E3 | 0.750 | 0.015 | 48.916 | 0.000 |
| **Formative Construct** | **Item** | **Weight** | **Variance Inflation Factor**  **(VIF)** |  |  |
| **Risk Perception** | R1 | 0.039 | 1.202 |  |  |
|  | R2 | 0.186 | 1.169 |  |  |
|  | R3 | 0.005 | 1.196 |  |  |
|  | R4 | 0.138 | 1.250 |  |  |
|  | R5 | 0.141 | 1.365 |  |  |
|  | R6 | 0.465 | 1.497 |  |  |
|  | R7 | 0.436 | 1.552 |  |  |
|  | R8 | 0.033 | 1.332 |  |  |
|  | R9 | 0.021 | 1.329 |  |  |
| **Knowledge** | K1 | 0.243 | 1.143 |  |  |
|  | K2 | 0.092 | 1.115 |  |  |
|  | K3 | 0.327 | 1.061 |  |  |
|  | K4 | 0.736 | 1.213 |  |  |
| **Official Communication** | O1 | 0.836 | 1.055 |  |  |
|  | O2 | 0.390 | 1.055 |  |  |

The SEM results (Figure S1) support all of the theoretical hypotheses that guided the selection of the above measurments in the study. All of the path coefficients tested in the model are statistically significant (p<0.001, Table S3).


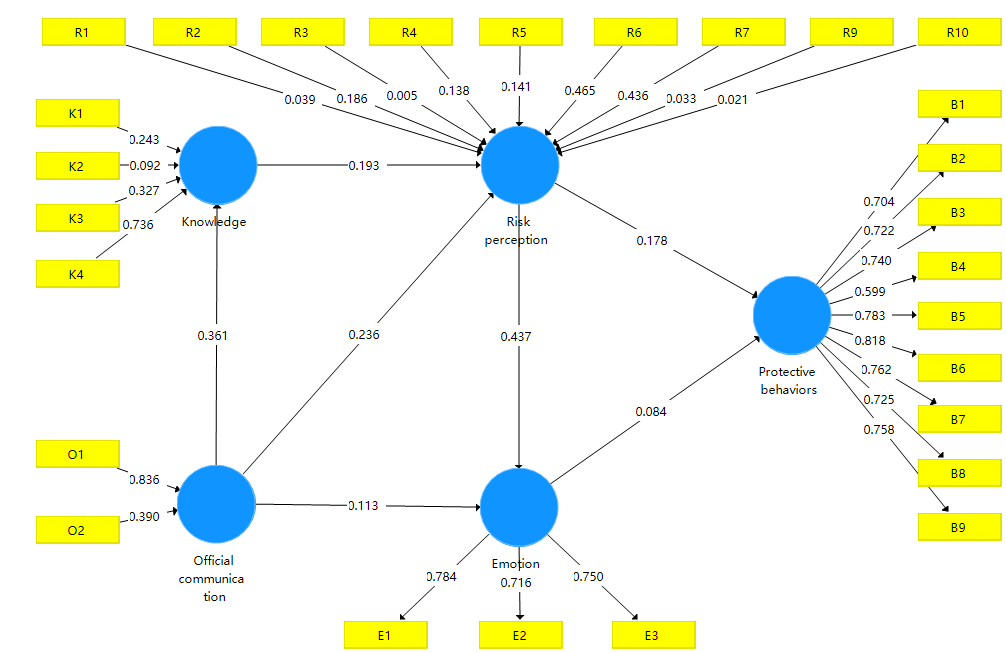


**Figure S1. Results of SEM**

**Table S3.** Path coefficients tested in the SEM

| **Path** | **Original Sample**  **(O)** | **Mean** | **Standard  Deviation** | **T** | **P** |
| --- | --- | --- | --- | --- | --- |
| Emotion -> Protective behaviors | 0.084 | 0.084 | 0.022 | 3.898 | 0.000 |
| Knowledge -> Risk perception | 0.193 | 0.193 | 0.031 | 6.183 | 0.000 |
| Official communication -> Emotion | 0.113 | 0.112 | 0.020 | 5.756 | 0.000 |
| Official communication -> Knowledge | 0.361 | 0.361 | 0.026 | 14.012 | 0.000 |
| Official communication -> Risk perception | 0.236 | 0.238 | 0.024 | 9.762 | 0.000 |
| Risk perception -> Emotion | 0.437 | 0.437 | 0.023 | 19.392 | 0.000 |
| Risk perception -> Protective behaviors | 0.178 | 0.180 | 0.026 | 6.979 | 0.000 |
