# Supplementary material for: The impacts of knowledge, risk perception, emotion and information on citizens’ protective behaviors during the outbreak of COVID-19: a cross-sectional study in China
Source: BMC Public Health. 2020 Nov 23;20:1751. doi: 10.1186/s12889-020-09892-y (PMC7681179; doi:10.1186/s12889-020-09892-y)
Supplement: Supplementary file 3 — Additional file 3: Table S4. Results of linear regression models [file 12889_2020_9892_MOESM3_ESM.docx]

**Additional file 3：**

**Table S4.** Results of linear regression models

**Table S4.** Results of linear regression models

| **Variable** | **Unstandardized Coefficients** | **(95% confidence interval)** | | **Standardized Coefficient Beta** | **t** | ***p*** | **Collinearity statistics** | |
| --- | --- | --- | --- | --- | --- | --- | --- | --- |
|  | **B** | **Lower Bound** | **Upper Bound** |  |  |  | [**Tolerance**](file:///D:\%25E6%259C%2589%25E9%2581%2593%25E8%25AF%258D%25E5%2585%25B8\Dict\8.9.3.0\resultui\html\index.html#javascript:;) | **VIF** |
| **(constant)** | 25.304 | 23.754 | 26.854 |  | 32.018 | 0.000 |  |  |
| **Gender (Female)** | 0.448 | -0.049 | 0.945 | 0.032 | 1.768 | 0.077 | 0.965 | 1.037 |
| **Age (years)** | 0.888 | 0.395 | 1.382 | 0.090 | 3.529 | 0.000 | 0.487 | 2.054 |
| **Education (University)** | -0.211 | -0.752 | 0.330 | -0.014 | -0.766 | 0.444 | 0.926 | 1.080 |
| **Marital status (Married)** | -0.410 | -1.093 | 0.273 | -0.029 | -1.177 | 0.239 | 0.499 | 2.003 |
| **Area (High-risk)** | -0.070 | -0.592 | 0.452 | -0.005 | -0.263 | 0.792 | 0.975 | 1.026 |
| **Knowledge (without incorrect answers)** | 1.758 | 1.245 | 2.272 | 0.121 | 6.709 | 0.000 | 0.970 | 1.031 |
| **Perceived susceptibility (>3)** | -0.300 | -1.165 | 0.566 | -0.012 | -0.679 | 0.497 | 0.961 | 1.041 |
| **Perceived severity (>3)** | 1.512 | 0.892 | 2.132 | 0.091 | 4.782 | 0.000 | 0.866 | 1.155 |
| **Perceived controllability (>3)** | 0.122 | -0.400 | 0.643 | 0.008 | 0.457 | 0.647 | 0.920 | 1.087 |
| **Negative emotion (>3)** | 0.511 | -0.107 | 1.128 | 0.030 | 1.622 | 0.105 | 0.902 | 1.108 |
| **Self-reported health status (>3)** | 2.178 | 1.626 | 2.730 | 0.140 | 7.738 | 0.000 | 0.964 | 1.038 |
| **Trust in official media (>3)** | 1.937 | 1.153 | 2.721 | 0.089 | 4.843 | 0.000 | 0.928 | 1.077 |
| **Information attention (>3)** | 4.955 | 3.778 | 6.131 | 0.153 | 8.259 | 0.000 | 0.913 | 1.095 |
